# Enhancement of In Vivo Bone Regeneration by the Carbohydrate Derivative DP2

**DOI:** 10.3390/ph18020215

**Published:** 2025-02-05

**Authors:** Nissrine Ballout, Sylvestre Toumieux, Walaa Darwiche, Cathy Gomila, Eric Trécherel, Franck Accadbled, Sara Laurencin-Dalicieux, Isabelle Gennero, José Kovensky, Agnès Boullier, Jérôme Ausseil

**Affiliations:** 1 Société d’Accélération du Transfert de Technologie-Nord, F-59800 Lille, France; 2 Institut Toulousain des Maladies Infectieuses et Inflammatoires, INSERM UMR1291, CNRS UMR5051, University of Toulouse, F-31024 Toulouse, France; 3 Service de Biochimie, Institut Fédératif de Biologie, CHU Toulouse, F-31024 Toulouse, France; 4 Laboratoire de Glycochimie et des Agroressources d’Amiens, UR 7378, Université de Picardie Jules Verne, F-80039 Amiens, France; sylvestre.toumieux@u-picardie.fr (S.T.); jose.kovensky@u-picardie.fr (J.K.); 5 Hematim Laboratory, EA4666, Université de Picardie Jules Verne, F-80054 Amiens, France; 6 Mécanismes Physiopathologiques et Conséquences des Calcifications Cardiovasculaires, UR7517, Centre Universitaire de Recherche en Santé, Université de Picardie Jules Verne, F-80054 Amiens, France; trecherel.eric@chu-amiens.fr (E.T.);; 7 Service d’Orthopédie, Hôpital des Enfants, CHU de Toulouse, F-31024 Toulouse, France; 8 Periodontology Department, CHU de Toulouse, F-31024 Toulouse, France; 9 CERPOP (Center for Epidemiology and Research in POPulation Health), Toulouse University, INSERM, Paul Sabatier University, F-31000 Toulouse, France; 10 Laboratory of Biochemistry, CHU Amiens-Picardie, F-80054 Amiens, France

**Keywords:** DP2, bone morphogenetic protein, osteoblast, bone regeneration, calvarial defect, biomaterial

## Abstract

**Background/Objectives**: Delays in bone healing and complications of remodeling constitute a major medical problem—particularly in older adults and patients with comorbidities. Current therapeutic approaches are based on strategies that promote bone regeneration. We recently identified a disaccharide compound (DP2) that enhances in vitro mineralization in human osteoblast cells via the early activation of Runx2 and the induction of osteoblast differentiation. **Methods**: First, a calcium quantification assay was performed to assess mineralization in MC3T3-E1 cells. Next, microcomputed tomography and histological analyses were used to examine in vivo bone repair in a rat 5 mm cranial defect model following the implantation of DP2 coupled to a micro/macroporous biphasic CaP ceramic (MBCP^+^) or collagen scaffold. **Results**: Here, we demonstrated that DP2 induced osteogenic differentiation and significantly elevated calcium matrix deposition in the murine preosteoblast cell line MC3T3-E1. We found that treatment with DP2 coupled to MBCP^+^ repaired the calvarial defect on post-implantation day 91. It significantly increased bone mineral density starting on day 29 post-treatment. In addition, DP2 did not induce ectopic bone formation. **Conclusions**: Taken as a whole, these results show that DP2 is a promising candidate treatment for delayed bone healing.

## 1. Introduction

Bone healing problems associated with compound fractures, pathological fractures, chronic steroid use, and diseases such as osteoporosis, osteopenia, and cancer can have a major impact on the quality of life in general and in older adults in particular [1]. Furthermore, delayed fracture healing increases the risk of malunion, nonunion, and secondary fractures and also imposes a substantial economic burden on healthcare systems and society [2]. Thus, accelerated bone healing can improve the quality of life of patients and reduce healthcare costs.

Bone grafting (with autografts or allografts) is the standard surgical strategy for the treatment of non-union [3]. However, these strategies have several limitations, such as donor site morbidity, chronic pain, high costs, and immune-mediated tissue rejection [4]. Therefore, tissue engineering using a combination of biomaterials and growth factors or stem cells has been developed as an alternative to improve bone healing [5]. Overall, bone regeneration is characterized by (i) osteogenesis (the process by which bone tissue is produced within cells) [6]; (ii) osteoinduction (a molecule’s ability to induce the biochemical cascade of cellular differentiation required for the formation of the bone matrix) [7]; and (iii) osteoconduction (a biomaterial’s ability to serve as passive support for bone growth) [8]. Besides being osteoconductive and/or osteoinductive, biomaterials must be biocompatible and biodegradable to avoid a host immune response and to decompose when new bone is formed [9]. Currently, several ceramic, polymeric, or composite biomaterials that mimic bone structure and composition are available for use in clinical practice [10,11].

Beyond the use of biomaterials as scaffolds, it has been shown that a range of osteoinductive compounds effectively promotes bone regeneration and healing [12]. These compounds include growth factors like bone morphogenetic proteins (BMPs) [12]. Indeed, combinations of collagen with BMP-2 or BMP-7 have been approved for orthopedic applications [13,14,15]. However, harmful side effects and complications have been reported in some patients [16,17,18,19,20]. Furthermore, the production of BMP-2 and BMP-7 is expensive and not readily scalable, which has limited the products’ clinical application [21].

Studies on disaccharide compounds that specifically promote bone regeneration are limited; however, research indicates that certain polysaccharides (chitosan and hyaluronic acid) play a significant role in bone tissue engineering [22]. In comparison to hydroxyapatite (HA) coatings, the mechanism of action of polysaccharides is based on stimulating cellular activities such as osteoblast proliferation, differentiation, and migration. Indeed, HA coatings enhance osteoconduction, improve osteointegration, and facilitate natural mineralization by providing structural support to the bone tissue [23].

We recently identified DP2 as a well-tolerated disaccharide compound obtained after convergent synthesis starting from glucose [24]. It stimulated osteoblast differentiation by inducing alkaline phosphatase activity and osteogenic markers expression, such as osteopontin and osteocalcin [24,25]. Interestingly, DP2 induced in vitro mineralization in human osteoblast cells (HOb) earlier than BMP-2; this was mainly due to earlier activation of Runx2 [25]. In the present study, we first confirmed the in vitro effect of DP2 on induced mineralization in pre-osteoblastic murine cells (the MC3T3-E1 cell line). We then used microcomputed tomography (µCT) and histological analyses to assess bone repair on post-implantation day 91 in a rat model of a 5 mm cranial defect loaded with DP2 coupled to micro/macroporous biphasic CaP ceramic (MBCP^+^) or collagen.

## 2. Results

### 2.1. DP2 Promotes Mineralization in MC3T3-E1 Cells

**Figure 1:**
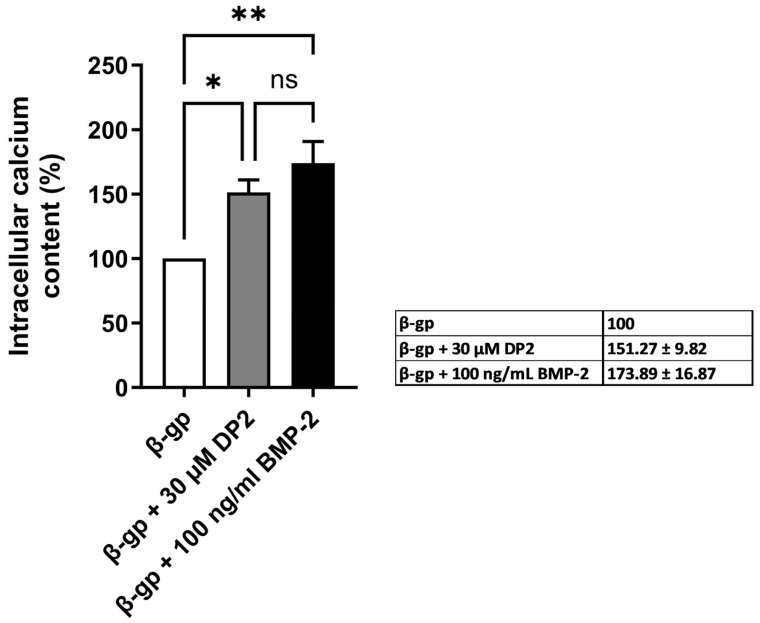
**Effect of DP2 on the mineralization of MC3T3-E1 cells** MC3T3-E1 cells were incubated with either 10 mM β-gp, 10 mM β-gp + 30 μM DP2, or 10 mM β-gp + 100 ng/mL BMP-2 for 25 days. Intracellular calcium was quantified using the OCP colorimetric method and normalized as a percentage of the value for cells treated with β-gp alone 100 (n = 4). Data are expressed as the mean ± SEM. The *p*-value was determined using a one-way ANOVA, followed by Tukey’s test for multiple comparisons (* *p* < 0.05, ** *p* < 0.01, ns: non-significant).

A characteristic feature of biological mineralization is the release of calcium phosphate crystals [26]. We first checked that DP2 increased β-glycerophosphate (β-gp)–induced mineralization in vitro in this murine cell model by treating MC3T3-E1 cells with 10 mM β-gp and adding either 30 µM DP2 or 100 ng/mL BMP-2 for 25 days. In our previous study [25], 30 µM was the lowest concentration of DP2 that induced mineralization; the same concentration was therefore used in the present study. Results showed that both 30 μM DP2 and 100 ng/mL BMP-2 (the effective concentration determined in previous studies, refs. [27,28,29] produced greater mean ± SEM mineralization of MC3T3-E1 cells when compared with the β-gp mineralizing medium alone (151.25 ± 9.82%, *p* < 0.05; and 173.88 ± 16.86%, *p* < 0.01, respectively) (Figure 1). These results indicate that DP2 promotes osteoblast mineralization to the same extent as BMP-2.

### 2.2. DP2 Enhances Bone Regeneration in a Rat Calvarial Defect

To evaluate the in vivo osteoinductive properties of DP2, two 5 mm calvarial defects were made in the skulls of adult male rats. One defect was left empty (the control), and the other one was filled with scaffold alone (MBCP^+^ or collagen) or scaffold coupled to 14 µg DP2, 42 µg DP2, or 10 µg BMP-2. The comparator dose levels were selected based on the similar in vitro and in vivo studies of BMP-2 [29,30,31,32]. Bone regeneration was then assessed (using µCT) on post-implantation days 14, 29, 63 and 91.

All animals survived the duration of the study, and no complications were observed. After 91 days, there were no clinical signs of infection, hematoma, or necrosis at the defect site, indicating that DP2 did not have any toxic effects in vivo.

- Quantitative analysis of bone mineral density (BMD)

**Figure 2:**
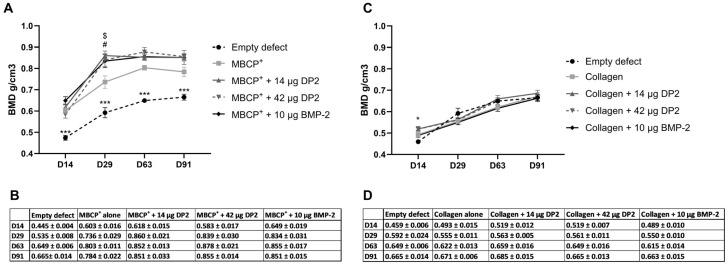
**Bone mineralization density (BMD, g/cm^3^) after implantation.** (**A**,**B**) Four groups of eight animals each were tested: MBCP^+^ alone, MBCP^+^ + 14 µg DP2, MBCP^+^ + 42 µg DP2, and MBCP^+^ + 10 µg BMP-2. BMD was calculated using CTAn software (version 1.14.4.1). Data are expressed as the mean ± SEM (n = 8). The *p*-value was determined in a one-way ANOVA, followed by Tukey’s test for multiple comparisons (*** *p* < 0.001 for the empty defect vs. all the other groups; $ *p* < 0.01 for MBCP^+^ alone vs. MBCP^+^ + 14 µg DP2; # *p* < 0.05 for MBCP^+^ alone vs. MBCP^+^ + 42 µg DP2) The table summarizes the results obtained for each group (g/cm^3^). (**C**,**D**) Four groups of eight animals each were tested: collagen alone, collagen + 14 µg DP2, collagen + 42 µg DP2, and collagen + 10 µg BMP-2. BMD was calculated using CTAn software. Data were expressed as the mean ± SEM (n = 8). The *p*-value was determined in a one-way ANOVA, followed by Tukey’s test for multiple comparisons (* *p* < 0.05 for the empty defect vs. collagen + 14 µg DP2 and for the empty defect vs. collagen + 42 µg DP2). The table summarizes the obtained results for each group (g/cm^3^).

Furthermore, all rats showed homogeneous bone reconstruction responses, as evaluated by calculating the values for the empty holes that served as an internal control for each rat. A quantitative analysis of bone mineral density (BMD) was performed using µCT. With the MBCP^+^ scaffold, a significant difference in the mean ± standard error of the mean (SEM) BMD between the empty defect (0.445 ± 0.004 g/cm^3^) and the other conditions was observed on day 14: MBCP^+^ alone (0.603 ± 0.016 g/cm^3^, *p* < 0.001), MBCP^+^ + 14 µg DP2 (0.618 ± 0.015 g/cm^3^, *p* < 0.001), MBCP^+^ + 42 µg DP2 (0.583 ± 0.017 g/cm^3^, *p* < 0.001), and MBCP^+^ + 10 µg BMP-2 (0.649 ± 0.019 g/cm^3^, *p* < 0.001). On day 29, the same significant differences between the empty defect (0.535 ± 0.008 g/cm^3^) and the other groups were observed. However, there was also a significant difference between the MBCP^+^ group (0.736 ± 0.029 g/cm^3^) on one hand and the MBCP^+^ + 14 µg DP2 group (0.860 ± 0.021 g/cm^3^, *p* < 0.01) and the MBCP^+^ + 42 µg DP2 group (0.839 ± 0.030 g/cm^3^, *p* < 0.05) on the other (Figure 2A,B). On days 63 and 91, the same trend as that on day 14 was observed for the various batches, which demonstrated the mineralization/maturation of the newly formed bone. With the collagen scaffold, a significant difference between the empty defect (0.459 ± 0.006 g/cm^3^) and the collagen + 14 µg DP2 group (0.519 ± 0.012 g/cm^3^, *p* < 0.05) and the collagen+ 42 µg DP2 group (0.519 ± 0.007 g/cm^3^, *p* < 0.05) was observed on day 14 (Figure 2C,D). However, from day 29 onwards, there were no intergroup differences.

- Quantitative analysis of bone volume fraction

**Figure 3:**
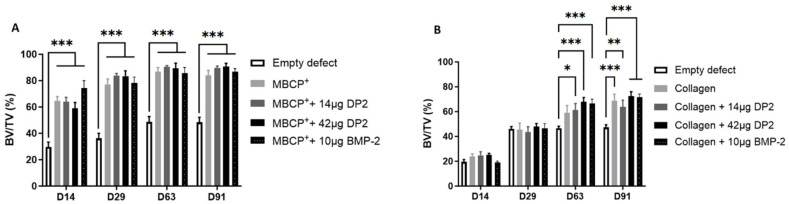
**Bone volume (BV/TV, %) after implantation.** (**A**) Four groups of eight animals were tested: MBCP^+^ alone, MBCP^+^ + 14 µg DP2, MBCP^+^ + 42 µg DP2, and MBCP^+^ + 10 µg BMP-2. (**B**) Four groups of eight animals were tested: collagen alone, collagen + 14 µg DP2, collagen + 42 µg DP2, and collagen + 10 µg BMP-2. BV/TV was calculated using CTAn software. Data were expressed as the mean ± SEM (n = 8). The *p*-value was determined using one-way ANOVA, followed by Tukey’s test for multiple comparisons (* *p* < 0.05, ** *p* < 0.01, *** *p* < 0.001).

The bone volume fraction (bone volume (BV)/tissue volume (TV), i.e., the volume of mineralized bone for a given volume of interest) was used as a quantitative indicator of the bone’s microstructure and strength. The results showed that with the MBCP^+^ scaffold, the BV/TV ratio was significantly higher in the different groups than in the empty defect. BV/TV peaked at 80% on day 29 and remained stable until day 91. However, there were no significant differences in BV/TV between the various MBCP^+^ groups (Figure 3A) or between the various collagen groups (Figure 3B).

**Figure 4:**
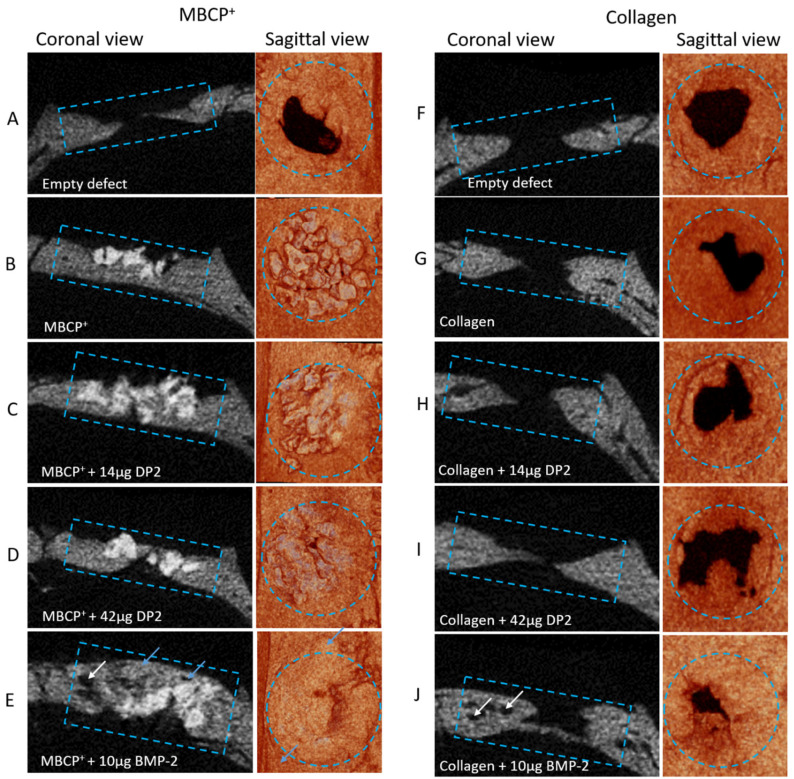
**Micro-CT images of calvarial defects on post-implantation day 91.** A coronal view (**left image**) and sagittal view (**right image**) of the defect are shown for each condition, and the images are representative of the animals in each group ((**A**–**F**): Empty defect, (**B**–**G**): scaffold alone, (**C**–**H**): scaffold + 14 µg DP2, (**D**–**I**): scaffold + 42 µg DP2, and (**E**–**J**): scaffold + 10 µg BMP-2). The blue rectangle and circle indicate the region of interest, the blue arrows represent ectopic bone, and the white arrows represent empty spaces between the original bone and the newly formed bone.

Analysis of the µCT images using CtVox software (version 3.3.0R1403; Figure 4) showed that new bone formation occurred in all groups. Coronal views indicated that bone bridging was thicker in all treatment conditions than in the empty defect. Ectopic reconstruction was observed in the MBCP^+^ + 10 µg BMP-2 condition, but not in the other conditions (Figure 4E). Moreover, in the collagen + 10 µg BMP-2 condition, the presence of small holes in the newly formed bone suggested the presence of a discontinuity with the original bone (Figure 4J).

- Histological analyses of newly formed bone

**Figure 5:**
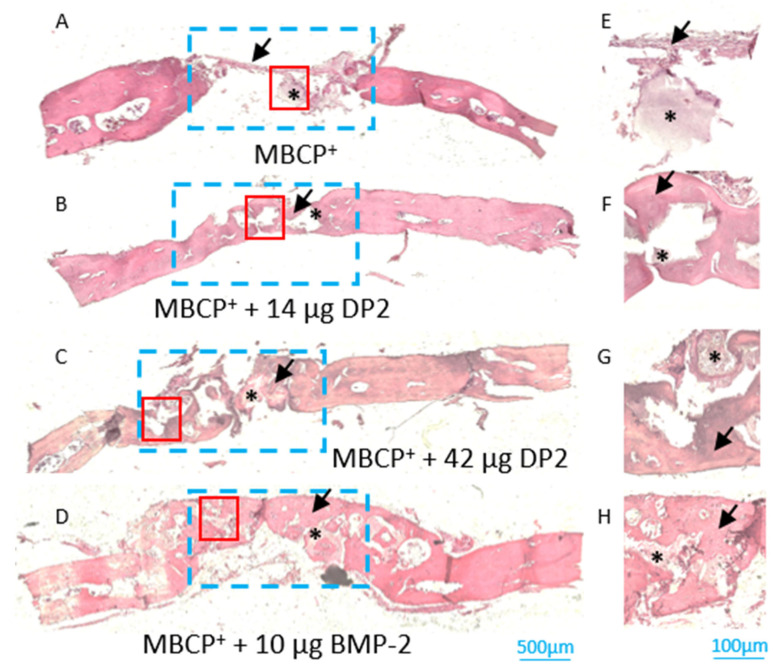
**Histologic assessments of calvarial defects (MBCP^+^ scaffold) on post-implantation day 91.** Bone sections were stained with H&E reagent. Black arrow: newly formed bone; black asterisk: residual MBCP^+^ scaffold. The blue rectangle represents the region of interest, and the red square represents the high-magnification insets E to H. (**A**–**E**) MBCP^+^ alone, (**B**–**F**) MBCP^+^ + 14 µg DP2, (**C**–**G**) MBCP^+^ + 42 µg DP2, and (**D**–**H**) MBCP^+^ + 10 µg BMP-2. Scale bars = 500 µm (**A**–**D**) or 100 µm (**E**–**H**).

**Figure 6:**
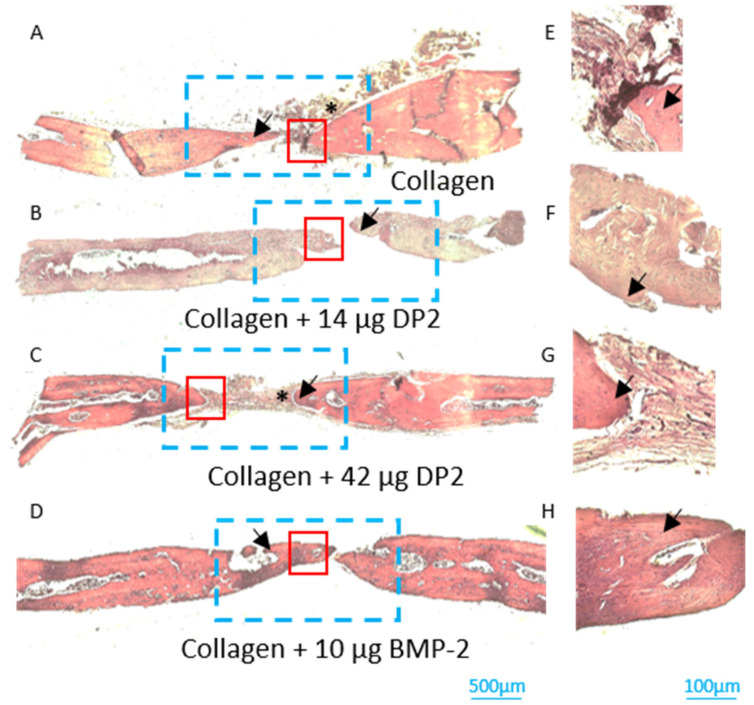
**Histologic assessments of calvarial defects (collagen scaffold) on post-implantation day 91.** Bone sections were stained with H&E reagent. Black arrow: newly formed bone; black asterisk: residual collagen matrix; the blue rectangle represents the region of interest; the red square represents the region the high-magnification insets E to H. (**A**–**E**) collagen alone, (**B**–**F**) collagen + 14 µg DP2, (**C**–**G**) collagen + 42 µg DP2, (**D**–**H**) and collagen + 10 µg BMP-2. Scale bars = 500 µm (**A**–**D**) or 100 µm (**E**–**H**).

After sacrifice on post-implantation day 91, the newly formed bone in the middle section of the defect was evaluated histologically. Histomorphometric analyses showed that bone reconstruction occurred in all four groups (black arrow, Figure 5 and Figure 6). However, the density of the newly formed bone was higher in the MBCP^+^ + 14 µg DP2, MBCP^+^ + 42 µg DP2, and MBCP^+^ + 10 µg BMP-2 conditions compared to MCBP^+^ alone. These results confirmed the µCT findings. As in the experiments with the collagen scaffold, histological analyses clearly confirmed the discontinuity between the original bone and newly formed bone. It should be noted that cells (osteoblasts and osteocytes) and vessels were observed in all batches and thus indicated that the bone was structurally mature. Residual scaffolds were also observed in most animals (black asterisk; Figure 5 and Figure 6).

## 3. Discussion

Bone defect repair and delayed fracture healing are major contributors to morbidity and mortality in older adults and, thus constitute a *serious health problem* [33,34]. Congenital pseudarthrosis of the tibia and the clavicle also constitute challenges in the field of pediatric orthopedics, and there is no consensus on the best treatment [35,36].

Currently, available treatments (autografts or allografts) can result in several complications and insufficient healing [37]. Growth factors are candidate treatments for fracture healing. However, due to the high cost of production and the occurrence of adverse events, the clinical use of growth factors has been limited. The growth factor BMP-2 has been studied extensively in the field of bone fracture healing but has been associated with adverse events (such as extreme inflammation and edema), poor quality bone regeneration, and uncontrolled ectopic bone formation [19,20,21], which was also found in the present study (Figure 4E). It is, therefore, essential to develop appropriate osteogenic molecules with a better safety profile and lower production costs.

We reported previously that DP2 could enhance mineralization in HObs [25]. Therefore, in the present study, we first confirmed the in vitro efficacy of DP2 in a murine pre-osteoblastic mineralization model (MC3T3-E1). Next, using a rat model of a 5 mm cranial defect loaded with DP2 coupled to MBCP^+^ or collagen, µCT, and histological analysis were used to examine the bone repair potential of DP2 relative to that of BMP-2. The in vitro results confirmed our findings with HObs and showed that DP2 increased mineralization induced by β-gp in MC3T3-E1 cells.

Furthermore, the results of many animal studies have shown that BMPs are efficacious in inducing bone mineralization [38]. However, this efficacy was dose-dependent, and the treatment response and extent of bone reconstruction varied from one species to another [39,40,41,42]. We, therefore, decided to test the osteoinductive properties of DP2 in a standard in vivo bone defect model: the cranial defect model in 8-week-old rats [43]. Two different dose levels of DP2 were tested (14 and 42 µg), coupled to a scaffold composed of MBCP^+^ (a bioactive calcium phosphate bone substitute) or collagen. The MBCP^+^ scaffold has already been tested in two European, multicenter, randomized clinical studies for bone regeneration [44,45]. We selected the dose level of DP2 used in this in vivo experiment by multiplying the concentration reported in similar in vitro bone regeneration studies in the literature by a factor of 100 or 300, taking into consideration the in vivo model used and the mode of administration [29,30,32,41].

Moreover, all rats showed homogeneous bone reconstruction responses, as evaluated by calculating the values for the empty holes that served as an internal control for each rat. Even though a significant increase in bone mineralization started on post-transplantation day 14, it was only on post-implantation day 29 that a significant difference was observed between the groups transplanted with the scaffold and the other groups. Importantly, the bone mineralization was higher in the MBCP^+^ + 14 µg DP2 group than in the MBCP^+^ + 42 µg DP2 and BMP-2 groups.

These results are consistent with our previous report, in which DP2 appeared to induce mineralization earlier than BMP-2 [25]. We have shown that DP2 is most effective at the level of osteoblast differentiation by enhancing the expression of the osteogenic transcription factor Runx2 earlier than BMP-2 and subsequently inducing the transcription of OCN, ColA1, and OPN [25]. It is noteworthy that we did not observe ectopic bone formation (an adverse event associated with BMPs) in the MBCP^+^ + DP2 group [46,47,48,49].

Moreover, it is well known that absorbable collagen scaffolds used with reconstituted BMP-2 retain a small percentage (around 10%) of the protein at the implant site. This means that a higher concentration of BMP-2 is required to achieve a therapeutic effect, resulting in irregular bone growth and a greater risk of adverse events [41,50,51,52]. Therefore, the effect of DP2 (14 µg or 42 µg) coupled to collagen was tested in this study. According to the μCT results, there was a significant difference between the collagen + DP2 groups and the empty defect but not between the collagen-only group and the other groups—suggesting a low level of affinity between DP2 and the collagen scaffold. Furthermore, treatment with collagen + 10 µg BMP-2 did not result in continuous repair of the the old bone and newly formed bone.

In conclusion, the present study confirmed that DP2 induces cell mineralization. Most importantly, DP2 implantation improved in vivo bone mineralization in the absence of adverse events.

## 4. Materials and Methods

### 4.1. Chemical Synthesis of DP2

**Figure 7:**
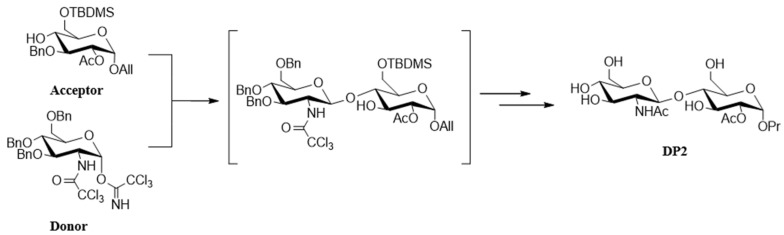
**Synthesis and structure of DP2.** The acceptor glucoside was obtained in eight steps, with total control over the alpha stereochemistry of the aglycone moiety. The activated alpha trichloroacetimidate donor glucoside holding a 2-aminotrichloroacetyl group was obtained in a straightforward, three-step sequence [24] and exclusively lead to the disaccharide with a beta configuration.

DP2 was obtained by a multistep chemical synthesis starting from commercially available glucose and glucosamine carbohydrates. Chemical modifications of each led to the two strategic compounds depicted here as donor and acceptor glycosides. This convergent synthesis of DP2 requires glycosylation of the acceptor glucoside with a glucoside donor [24]. The key glycosylation reaction is completely stereoselective, and after the deprotection steps, it affords the DP2 disaccharide as a pure single diastereoisomer (Figure 7).

### 4.2. Cell Culture

The murine preosteoblast cell line (MC3T3-E1, originally isolated from a mouse pup) was purchased from the American Type Culture Collection (ATCC, Manassas, VA, USA). The cells were incubated at 37 °C in a 5% CO_2_ atmosphere with 90% humidity. MC3T3-E1 cells were cultured in 75 cm^2^ flasks containing 15 mL of α-MEM (M4526, Sigma-Aldrich, MO, USA) with 10% fetal calf serum (FCS, PAN-Biotech GmbH, Aidenbach, Germany), supplemented with 1% penicillin/streptomycin (Sigma-Aldrich, St. Louis, MO, USA) and 1% glutamine (200 mM, Sigma-Aldrich, St. Louis, MO, USA). To induce mineralization, MC3T3-E1 cells were treated with α-MEM with 5% FCS, supplemented with 10 mM β-gp (216.04 g/mol, Sigma-Aldrich, MO, USA) and 50 µg/mL ascorbic acid (176.12 g/mol, Sigma-Aldrich, St. Louis, MO, USA). BMP-2 (100 ng/mL; Biotechne, Minneapolis, MN, USA) or DP2 (30 µM) was then added. In a pilot study, HObs were treated with various concentrations of DP2 (from 5 to 50 µg); the results showed that mineralization was first detected at a concentration of 30 µM DP2. The tested concentration of BMP-2 (100 ng/mL) has been used in several similar studies in the literature [27,28,29].

### 4.3. Calcium Quantification Assay

The intracellular calcium content was assessed using the o-cresolphthalein complexone assay (OCP), as described previously [53]. Briefly, cells were seeded in 48-well plates at 5000 cells/well. On post-treatment day 25, cells were washed several times with PBS and decalcified with 300 μL 0.6 N HCl per well for 2 h at room temperature. The calcium content of the HCl supernatant was determined colorimetrically (optical density at 565 nm) using an OCP assay. The intracellular calcium content was normalized to the protein content, as determined using the Peterson assay.

### 4.4. Animals and Surgical Procedures

**Figure 8:**
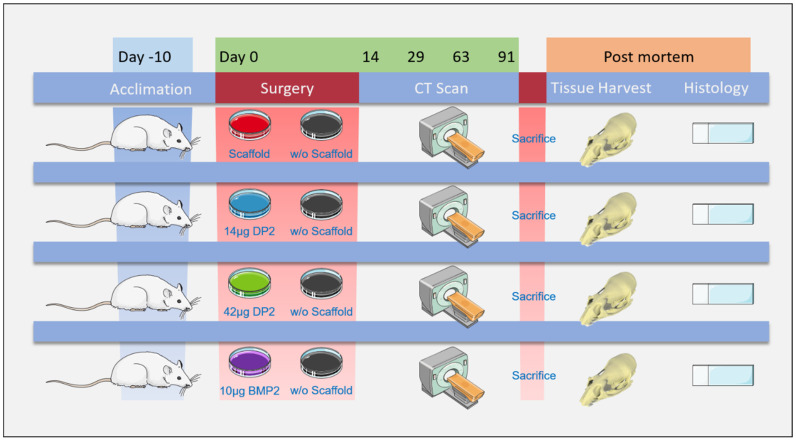
**The experimental protocol.** Two 5 mm diameter cranial lesions were created on each side of the sagittal suture using a diamond bur. Eight groups of animals (n = 8 each) were treated with (i) MBCP+ alone, (ii) MBCP^+^ + 14 µg DP2, (iii) MBCP^+^ + 42 µg DP2, (iv) MBCP^+^ + 10 µg BMP-2, (v) collagen alone, (vi) collagen + 14 µg DP2, (vii) collagen + 42 µg DP2, or (viii) collagen + 10 µg BMP-2. Each scaffold was implanted on the left side; the right side remained empty and served as an internal control. A µCT scan was performed on post-implantation days 14, 29, 63, and 91. On post-implantation day 91, the rats were euthanized with CO2. The region of interest comprising the two calvarial defects was then harvested for histological analysis.

All experimental procedures (Figure 8) were approved by the local animal care and use committee and by the French Ministry of Research (reference: APAFIS#15464-2018060810566765). Sixty-four 8-week-old male Sprague-Dawley rats (Janvier Labs, Le Genest-Saint-Isle, France) were housed in a facility with a 12-h day-night cycle, a temperature of 22 ± 2 °C, and 50% humidity. The animals were given free access to water and food and were weighed regularly.

In order to assess DP2’s osteoinductive activity and determine which of the two dose levels was more efficacious, two bone defects were created in the calvaria of adult rats. BMP-2 was used as a reference. DP2 or BMP-2 was coupled to MBCP^+^ (Biomatlante, Nantes, France), a 20:80 (± 10) mixture of hydroxyapatite and beta-tricalcium phosphate (β-TCP), used as a scaffold in bone tissue engineering. Since collagen is the scaffold used in clinical applications of BMP-2, we also tested it as a scaffold for DP2 (cell-culture-grade collagen C9879, Sigma-Aldrich, MO, USA).

Rats were anesthetized with isoflurane (IsoVet, Centravet, Amiens, France): 5% at 1 L/min for induction and 2 to 3% at 0.5 L/min for maintenance. After the skin was shaved, a sagittal incision was made, and the periosteum was dissected. Two 5 mm diameter cranial lesions (one on each side of the sagittal suture) were made with a diamond bur [54]. Eight groups of eight animals were tested with (i) MBCP^+^ alone, (ii) MBCP^+^ + 14 µg DP2, (iii) MBCP^+^ + 42 µg DP2, (iv) MBCP^+^ + 10 µg BMP-2, (v) collagen alone, (vi) collagen + 14 µg DP2, (vii) collagen + 42 µg DP2, and (viii) collagen + 10 µg BMP-2. The scaffolds were implanted on the left side, and the right side was left empty as an internal control for the effects of the injury and natural repair processes in the absence of a scaffold. The periosteum and the skin were sutured. Subcutaneously administered buprenorphine (0.05 mg/kg) was used as an analgesic after surgery, and the animals’ welfare was monitored daily.

### 4.5. MicroCT Analysis

In order to monitor bone formation, a µCT scan was performed on post-implantation days 14, 29, 63, and 91. During the µCT scan, the rats were anesthetized with isoflurane (5% at 1 L/min for induction and 2 to 3% at 0.5 L/min for maintenance). Animals were placed in a scanner (SkyScan 1176 microCT; Bruker, Billerica, MA, USA) [55]. The scanner parameters were as follows: Alu-1 mm filter, voltage 65 kV, intensity 380 μA, pixel size 18 µm, and rotation 0.6°. The scan lasted between 5 and 6 min for each animal. When the scan was complete, anesthesia was stopped, and the animal was removed from the device and returned to its standard housing conditions. Three-dimensional reconstructions were obtained using NRecon software (version 1.7.4.6, Bruker, Billerica, MA, USA). A Gaussian filter was used to reduce the level of background noise. The other reconstruction parameters were as follows: ring artifact 8, Beam 36%, Output between 0 and 0.062. Three-dimensional renderings were extracted from the data frames using Dataviewer software (version 1.5.6.2, Bruker, Billerica, MA, USA). The bone mineralization density (BMD, in g/cm^3^ of hydroxyapatite), BV, and TV were obtained using CTAn software (version 1.14.4.1, Bruker, Billerica, MA, USA). Three-dimensional photos were processed using CTVox software (version 3.3.0R1403, Bruker, Billerica, MA, USA).

### 4.6. Tissue Preparation and Histological Analyses

In order to study the cell composition, tissue morphology, and phenotype, we performed histologic and immunohistochemical assessments. On post-implantation day 91, the rats were euthanized with CO_2_, and the region of interest comprising the two calvarial defects was harvested. The tissue was fixed with 4% paraformaldehyde for 48 h and then decalcified in a 10% EDTA solution for a total of 4 weeks (the solution was changed every week), with continuous stirring. The tissue was then embedded in paraffin (Tissue-Tek VIP^®^ 6 AI, Sakura Finetek, Villeneuve-d’Ascq, France), and 7 µm sagittal sections were prepared with a microtome (RM2255, Leica, Wetzlar, Germany). For hematoxylin and eosin (H&E) staining, the sections were washed twice with xylene, once with 100% ethanol (for 1 min), and then successively with 100%, 95%, and 70% ethanol and water for 3 min each. Sections were incubated with Mayer’s Hematoxylin Solution (Sigma-Aldrich, St. Louise, MO, USA) and then rinsed for 5 min with water. After incubation with 2% eosin (624.06 g/mol, Sigma-Aldrich, St. Louise, MO, USA), sections were rinsed with water. The sections were dehydrated by incubation for 5 min with 95% ethanol and then twice for 5 min with 100% ethanol. After rapid washing with xylene, a drop of mounting medium (DPX, Sigma-Aldrich, MO, USA) and a coverslip were placed on the slide. After drying, the sections were examined for newly formed bone under the objective (x20) of a light microscope (Observer Z1, Zeiss, Oberkochen, Germany).

### 4.7. Statistical Analysis

Data for quantitative variables are expressed as mean ± SEM. All statistical analyses were performed using GraphPad Prism software (version 9.00, GraphPad Software, Boston, MA, USA). Depending on whether the data were normally distributed (according to a Shapiro-Wilk test), groups were compared using the Mann-Whitney test, Wilcoxon signed-rank test, one-way analysis of variance (ANOVA) followed by the Kruskal-Wallis test, or two-way ANOVA followed by Tukey’s test for multiple comparisons. The threshold for statistical significance was set at *p* < 0.05.

## 5. Conclusions

Bone defect repair and delayed fracture healing impose a substantial economic burden on healthcare systems and society and can have a major impact on the quality of life in general and in older adults in particular. Thus, accelerated bone healing can improve the quality of life for patients and reduce healthcare costs. Currently available treatments (autografts or allografts) can result in several complications and insufficient healing. Growth factors are candidate treatments for fracture healing. However, due to the high cost of production and the occurrence of adverse events, the clinical use of growth factors has been limited. It is, therefore, essential to develop appropriate osteogenic molecules with a better safety profile and lower production costs.

In this study, we identified DP2 as a well-tolerated disaccharide compound obtained after convergent synthesis starting from glucose. We confirmed the in vitro effect of DP2 on induced mineralization in pre-osteoblastic murine cells (MC3T3-E1 cell line) and improved in vivo bone mineralization. Consequently, DP2 should be considered a drug candidate for the treatment of delayed bone fracture repair. It might be possible to use DP2 for orthopedic indications in adults (such as osteotomy and pseudarthrosis) and in children (such as congenital pseudarthrosis of the tibia and clavicle). DP2 can also promote faster bone wound healing processes, particularly in patients with compromised bone healing capacities, or when fibroblastic wound healing competes with osteoblastic healing, particularly in the oral cavity (such as alveolar bone healing). As DP2 is water-soluble, it needs to be injected into hydrogels or scaffolds for bone tissue engineering. Thus, it would be interesting to combine the biomaterial “DP2-hydrogel” with autografts for patients with comorbidities, such as diabetes, smoking, and anemia.

## Data Availability

The data presented in this study are available upon request from the corresponding author. The authors confirm that all relevant data are included in this article.
